# Shifting Thresholds: Changes in Antihypertensive Eligibility Under the 2025 Versus 2017 Hypertension Guidelines

**DOI:** 10.1016/j.jacadv.2025.102546

**Published:** 2026-01-21

**Authors:** Faith E. Metlock, Bede N. Nriagu, Britton Scheuermann, Carl Ade, Yaa Adoma Kwapong, Alexander C. Razavi, Stephen Juraschek, Sharmaine M. McCoy, Lily N. Dastmalchi, Garima Sharma, Jared A. Spitz

**Affiliations:** a Inova Schar Heart and Vascular, Inova Health System, Falls Church, USA; b College of Health and Human Sciences, Kansas State University, Manhattan, USA; c Johns Hopkins School of Medicine, Baltimore, USA; d Emory Clinical Cardiovascular Research Institute, Emory University School of Medicine, Atlanta, USA; e Beth Israel Deaconess Medical Center, Harvard Medical School, Boston, USA

**Keywords:** antihypertensive therapy, blood pressure guidelines, cardiovascular disease risk, hypertension, PREVENT equations

## Abstract

**Background:**

Hypertension affects nearly half of U.S. adults. The 2025 American College of Cardiology/American Heart Association guideline adopts the Predicting Risk of Cardiovascular Disease Events (PREVENT) risk equations and updates treatment recommendations for stage 1 hypertension, potentially altering eligibility for antihypertensive therapy.

**Objectives:**

The primary objective was to quantify changes in antihypertensive treatment eligibility under the 2025 vs 2017 guidelines. Secondary objectives were to characterize adults newly meeting treatment thresholds, assess concordance and discordance in eligibility, and evaluate robustness across PREVENT model variants.

**Methods:**

We conducted a simulation-based analysis using nationally representative National Health and Nutrition Examination Survey data (2017-2020) among adults aged 30 to 79 years. Treatment eligibility was assigned using 2017 and 2025 guideline criteria. Survey-weighted estimates quantified population-level eligibility, newly eligible adults, and concordance patterns. Analyses were repeated using PREVENT Base, Full, hemoglobin A1c, and albumin-to-creatinine ratio variants, and multivariable models identified predictors of eligibility.

**Results:**

Among 5,578 adults (weighted population 160 million), 36.4% were eligible for treatment under the 2017 guideline and 36.6% under the 2025 guideline, representing a minimal net increase of 0.7% (approximately 400,000 adults). Most adults were consistently ineligible (63.3%), whereas one-third were consistently eligible (36.3%). Newly eligible adults were predominantly older women with higher body mass index and borderline glycemic measures but without established cardiovascular disease. Eligibility patterns were stable across racial and ethnic groups. Analyses were repeated across all PREVENT risk equation variants, and multivariable models identified predictors of eligibility.

**Conclusions:**

Adoption of the 2025 American College of Cardiology/American Heart Association guideline results in a minimal expansion of antihypertensive treatment eligibility. Results were robust across PREVENT model variants, supporting risk-based guideline implementation.

Hypertension affects nearly half of adults in the United States and is projected to increase substantially in prevalence and impact over the next 25 years, leading to increased risks in cardiovascular morbidity and mortality.^1^ It remains the leading modifiable risk factor for cardiovascular disease (CVD), driving coronary heart disease, stroke, and heart failure, and is a major contributor to premature death.^2^ Despite effective therapies, blood pressure awareness, treatment, and control remain unacceptably low, with particularly poor outcomes among minoritized racial/ethnic adults and those living in adverse social and economic conditions.^3^^,^^4^ These disparities highlight the need for prevention and treatment strategies that more accurately and equitably identify those at the highest risk.

Clinical guidelines have provided the framework for hypertension management by defining thresholds for treatment initiation. The 2017 American College of Cardiology (ACC)/American Heart Association (AHA) guideline recommended pharmacologic therapy for adults with blood pressure ≥140/90 mm Hg, or for those with stage 1 hypertension (130-139/80-89 mm Hg) who either had diabetes, chronic kidney disease, or a 10-year predicted atherosclerotic CVD (ASCVD) risk ≥10% using the Pooled Cohort Equations (PCE).^5^, ^6^, ^7^ More recently, the AHA introduced the Predicting Risk of CVD Events (PREVENT) equations to address limitations of prior risk tools, including restricted outcome definitions and reliance on race as a predictor.^6^^,^^8^, ^9^, ^10^ The 2025 AHA/ACC Hypertension Guideline formally incorporated PREVENT into treatment recommendations.^11^ Under this framework, adults with blood pressure ≥140/90 mm Hg remain eligible for pharmacologic therapy, whereas those with stage 1 hypertension are recommended for therapy if they have clinical CVD (including heart failure), diabetes, chronic kidney disease, or a PREVENT-predicted 10-year CVD risk ≥7.5%. Prior work has demonstrated that guideline changes can substantially alter the size and composition of the treatment-eligible population, with direct implications for prevention and equity.^12^^,^^13^ However, it is unknown how adoption of PREVENT will reshape treatment candidacy in the United States, particularly which adults will become newly eligible and what clinical and social characteristics distinguish them. To address this gap, we used nationally representative data from the National Health and Nutrition Examination Survey (NHANES) 2017 to 2020 to compare antihypertensive treatment eligibility under the 2017 and 2025 guidelines, characterize adults newly meeting treatment thresholds, and identify clinical and sociodemographic predictors of eligibility under the updated framework (Central Illustration).

**Central Illustration:**
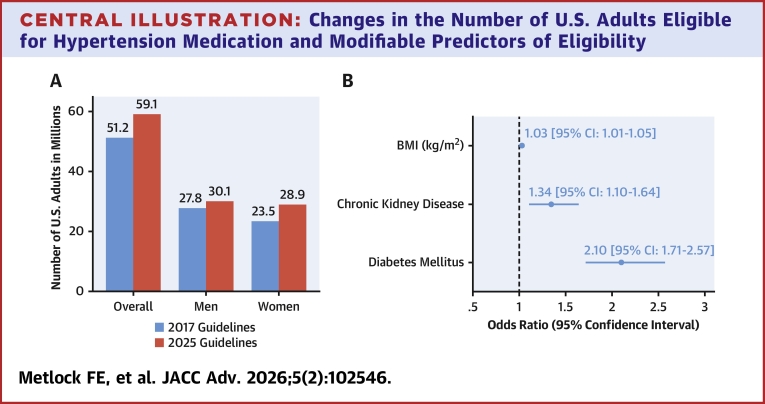
**Changes in the Number of U.S. Adults Eligible for Hypertension Medication and Modifiable Predictors of Eligibility** (A) Number of U.S. adults recommended pharmacological treatment of hypertension according to the 2017 and 2025 AHA/ACC guidelines. (B) Adjusted ORs (95% CI) are shown for notable clinical predictors of eligibility under the 2025 AHA/ACC blood pressure guideline, using the PREVENT Base 10-year risk equation. Models were adjusted for age, sex, race/ethnicity, education, employment, insurance, marital status, body mass index, diabetes, and chronic kidney disease. BMI = body mass index.

## Methods

### Study cohort

This investigation used publicly available data obtained from the NHANES, a cross-sectional sample of the civilian noninstitutionalized U.S. general population.^14^ We conducted a simulation-based analysis to estimate the proportion of U.S. adults meeting guideline-based thresholds for initiation of antihypertensive therapy under the 2017 and 2025 AHA/ACC hypertension guidelines.^5^^,^^11^ For the current analysis, we used data from the 2017 to 2020 NHANES cycles. Persons younger than 30 years or older than 79 years, those with missing blood pressure, incomplete data on self-reported antihypertensive medication, or data required for PCE and PREVENT risk equations were excluded. Due to differing clinical guidelines for blood pressure among pregnant individuals, those who were pregnant at the time of the survey were also excluded from the analysis. The continuous NHANES study was reviewed and approved by the U.S. National Center for Health Statistics Institutional Review Board, and all participants provided informed written consent. Supplemental Figure 1.

### Arterial blood pressure and thresholds for treatment

Systolic and diastolic arterial blood pressures were obtained in triplicate at 30-second intervals during the NHANES physical examination after a 5-minute seated resting period. The mean average was used to define systolic blood pressure and diastolic blood pressure. The current use of prescribed antihypertensive medication was defined by self-report. The eligibility for initiation of medical therapy was assessed according to the criteria outlined in the published 2017 ACC/AHA^5^ and 2025 AHA/ACC^11^ guidelines and refers to meeting guideline-based treatment thresholds rather than treatment access or receipt, based on history of CVD (coronary heart disease, stroke, heart failure), history of diabetes and chronic kidney disease, and high CVD risk, as outlined in Supplemental Table 1.

### CVD risk

The history of clinical CVD was defined by self-report of a prior diagnosis of coronary heart disease, myocardial infarction, stroke, or heart failure. Calculation of the PREVENT and PCE 10-year predicted CVD and ASCVD risk was performed using NHANES variables.^8^^,^^15^ Detailed interviews and physical assessment were used to obtain demographic information (age at the time of screening for the NHANES study, sex, and race), medical history, current/prior smoking history, and anthropometric measurements. Serum measurements of creatinine, glucose, total cholesterol, and high-density lipoprotein cholesterol were performed at a central laboratory. Estimated glomerular filtration rate was calculated using the Chronic Kidney Disease Epidemiology Collaboration 2021 creatinine equation.^16^^,^^17^ Diabetes mellitus was defined using the 2014 criteria established by the American Diabetes Association,^18^ a fasting plasma glucose ≥126 mg/dL and/or use of insulin or antihyperglycemic medications. Chronic kidney disease was defined as an estimated glomerular filtration rate <60 mL/min/1.73 m^2^.^19^

### Statistical analysis

All analyses herein accounted for the complex survey design of the NHANES study by adjusting for sample weights, clustering, and stratification (determined in concordance with NHANES guidelines) to obtain U.S. nationally representative estimates to account for oversampling and nonresponse with NHANES.^20^ Survey adjusted analyses were performed using the built-it *svy* survey prefix command in STATA (v.19.0, STATA Corp LLC). Continuous data are presented as mean and SDs or 95% CIs and categorical data are presented as count and percentage, unless otherwise noted.

The main outcome was to estimate the number and characteristics of adults eligible for hypertension treatment, with the exposure determined by the 2017 vs 2025 guidelines. Covariates were defined a priori based on their role in guideline eligibility and PCE/PREVENT risk stratification.^5^^,^^11^ Comparisons in demographics, socioeconomic characteristics, vital signs, and ASCVD risk predictions were conducted between those eligible under the 2017 or 2025 criteria using regression tests or Pearson chi-square tests, where appropriate. Subsequently, analyses were stratified to compare those newly eligible under 2025 guidelines to those eligible under both 2017 and 2025 guidelines. Differences in sample characteristics were also evaluated using concordant or discordant criteria, that is, whether a participant was eligible under both criteria or only eligible under 1 of the 2 guidelines. As sensitivity analyses, comparisons were reanalyzed under the enhanced PREVENT models using hemoglobin A1c (HbA1c) and urinary albumin-to-creatinine ratios (ACRs), as well as using the 30-year prediction models.

The 2 guidelines were then used to determine the projected differences in the numbers of U.S. adults eligible for hypertension treatment using Pearson chi-square tests. Subsequently, multivariate logistic regression was used for the identification of predictors that significantly influence the eligibility for hypertension treatment under the 2025 guidelines. Two separate binary outcomes were used: (1) whether or not an individual was eligible under 2025 guidelines (regardless of 2017 eligibility) and (2) whether or not an individual was *newly* eligible under 2025 guidelines (and therefore not eligible under the 2017 guidelines). The predetermined predictor variables entered into each model were age, sex, race/ethnicity, education level, marital status, family poverty-to-income ratio, health insurance, employment status, body mass index (BMI), total cholesterol, smoking status, chronic kidney disease, diabetes mellitus, the use of antihypertensive mediation, antihyperglycemic medication, and statin use. Collinearity was assessed in logistic regression models using variance inflation factors; a variance inflation factor <10 indicates no significant multicollinearity. This analysis was additionally repeated using the fully enhanced PREVENT 10-year model (using both HbA1c and urinary ACR, as social deprivation index is not available in NHANES).

All statistical procedures were run in STATA (version 19.5, STATA Corp LLC) with statistical significance set a priori at α = 0.05. Data were analyzed from August 22, 2025, through September 19, 2025.

## Results

### Study sample

The mean age of the study sample was approximately 52 years, with a balanced distribution by sex. Under both guidelines, adults meeting the treatment criteria were older and had higher BMI, blood pressure, fasting glucose, and HbA1c than those not meeting the criteria. Overall treatment eligibility was similar under the 2025 and 2017 guidelines, although the composition of eligible groups differed slightly, with the 2025 guideline identifying a somewhat younger and more female population. Racial and ethnic distributions were largely consistent across guidelines, with non-Hispanic Black adults disproportionately represented among treatment-eligible individuals. Eligible adults also had lower educational attainment and higher cardiometabolic risk profiles, including higher predicted 10-year CVD risk across both the PCE and PREVENT models Table 1.

**Table 1:** Demographic Characteristics of the Overall Population and by 2025 and 2017 Eligibility

	Overall	Only ACC/AHA 2017 Eligible^a^	New ACC/AHA 2025 Eligibility^b^	ACC/AHA 2017 and 2025 Eligible^c^	*P* Value
N survey-weighted (weighted %)^d^	160,704,463 (100.0%)	101,045 (0.1%)	522,699 (0.3%)	58,347,898 (36.3%)	
Sociodemographic					
Age, y (SD)	53 (13.3)	55 (6.0)	64 (3.051)	56 (12.9)	<0.001
Sex					0.02
Females, n (%)	81,186,283 (50.5%)	13,020 (12.9%)	457,773 (87.6%)	28,389,292 (48.7%)	
Males, n (%)	79,518,180 (49.5%)	88,025 (87.1%)	64,927 (12.4%)	29,958,606 (51.3%)	
Race/ethnicity					<0.001
Mexican American, n (%)	11,618,662 (7.2%)	0 (0.0%)	15,349 (2.9%)	3,641,412 (6.2%)	
Non-Hispanic Asian, n (%)	8,724,176 (5.4%)	17,843 (17.7%)	8,129 (1.6%)	3,206,854 (5.5%)	
Non-Hispanic Black, n (%)	16,533,171 (10.3%)	41,072 (40.6%)	82,424 (15.8%)	8,469,629 (14.5%)	
Non-Hispanic White, n (%)	105,312,262 (65.5%)	21,048 (20.8%)	403,673 (77.2%)	36,104,811 (61.9%)	
Other Hispanic, n (%)	11,513,673 (7.2%)	21,082 (20.9%)	13,125 (2.5%)	4,305,639 (7.4%)	
Other/multiracial, n (%)	7,002,518 (4.4%)	0 (0.0%)	0 (0.0%)	2,619,553 (4.5%)	
Education Level					<0.001
College graduate or above, n (%)	53,735,043 (33.5%)	30,863 (30.5%)	96,833 (18.5%)	14,844,897 (25.5%)	
High school graduate/General Educational Development Certificate or equivalent, n (%)	40,381,492 (25.1%)	17,410 (17.2%)	220,372 (42.2%)	17,305,465 (29.7%)	
Less than 9th grade, n (%)	17,145,675 (10.7%)	27,269 (27.0%)	66,580 (12.7%)	7,613,141 (13.1%)	
Some college or AA degree	49,376,426 (30.7%)	25,503 (25.2%)	138,915 (26.6%)	18,561,590 (31.8%)	
Marital status					0.025
Married/living with partner, n (%)	109,966,588 (68.4%)	83,570 (82.7%)	289,648 (55.4%)	38,996,549 (66.9%)	
Never married, n (%)	17,392,507 (10.8%)	0 (0.0%)	21,237 (4.1%)	5,793,972 (9.9%)	
Widowed/divorced/separated, n (%)	33,293,719 (20.7%)	17,475 (17.3%)	211,815 (40.5%)	13,539,021 (23.2%)	
Family income to poverty ratio, n (%)	109,966,588 (68.4%)	83,570 (82.7%)	289,648 (55.4%)	38,996,549 (66.9%)	0.010
Currently employed, n (%)	104,426,433 (65.0%)	59,030 (58.4%)	166,538 (31.9%)	34,518,770 (59.2%)	<0.001
Health insurance coverage, (%)	142,784,659 (89.0%)	79,997 (79.2%)	492,500 (94.2%)	51,921,786 (89.0%)	0.786
Biometrics					
BMI, kgm^2^ (SD)	30.3 (7.2)	29.3 (2.4)	32.9 (6.4)	31.1 (7.1)	<0.001
SBP, mm Hg (SD)	123.3 (16.9)	132.4 (4.3)	133.0 (5.5)	139.2 (14.9)	<0.001
DBP, mm Hg (SD)	75.2 (10.8)	82.4 (4.3)	76.2 (5.8)	83.8 (9.9)	<0.001
Blood glucose, mg/dL (SD)	112.3 (35.1)	97.3 (13.6)	104.2 (6.1)	123.9 (45.7)	<0.001
HbA1c	5.8 (1.0)	5.4 (0.5)	5.9 (0.3)	6.0 (1.2)	<0.001
Prevent equations					
PREVENT_BASE_10	8.5 (8.0)	6.5 (1.1)	8.7 (1.34)	12.4 (8.7)	<0.001
PREVENT_wACR_10	7.9 (8.0)	5.5 (1.1)	8.3 (1.6)	11.9 (9.3)	<0.001
PREVENT_wHbA1c_10	14.2 (12.3)	13.9 (2.1)	19.8 (2.7)	20.1 (13.1)	<0.001
PREVENT_Full_10	12.5 (11.9)	9.3 (2.0)	16.7 (3.2)	18.1 (13.2)	<0.001
PREVENT_BASE_30	25.9 (13.0)	28.6 (3.9)	31.8 (3.4)	33.7 (11.0)	<0.001
PREVENT_Full_30	30.2 (14.6)	33.1 (3.7)	42.0 (4.1)	38.6 (12.4)	<0.001
PCE_10 y^e^	10.0 (11.7)	11.6 (2.4)	7.3 (1.4)	15.9 (14.3)	<0.001
Prior ASCVD					
Yes	16,308,930 (10.1%)	7,129 (7.1%)	0 (0.0%)	9,028,177 (15.5%)	<0.001

ACC = American College of Cardiology; AHA = American Heart Association; ASCVD = atherosclerotic cardiovascular disease; BMI = body mass index; DBP = diastolic blood pressure; HbA1c = hemoglobin A1C; PCE = Pooled Cohort Equation; SBP = systolic blood pressure.

a Indicates adults only meeting antihypertensive treatment eligibility under the 2017 AHA/ACC guideline, which recommends therapy for: 1) systolic BP ≥ 140 mm Hg or diastolic BP ≥90 mm Hg; or 2) systolic BP 130 to 139 mm Hg or diastolic BP 80 to 89 mm Hg with diabetes, chronic kidney disease, clinical CVD, or a PCE-predicted 10-year ASCVD risk ≥10%.

b Indicates newly eligible adults under the 2025 AHA/ACC/Multi-Society guideline, which recommends therapy for: 1) systolic BP ≥140 mm Hg or diastolic BP ≥90 mm Hg; 2) systolic BP 130 to 139 mm Hg or diastolic BP 80 to 89 mm Hg in the presence of clinical CVD, diabetes, or chronic kidney disease; or 3) systolic BP 130 to 139 mm Hg or diastolic BP 80 to 89 mm Hg with a PREVENT-predicted 10-year total CVD risk ≥7.5%.

c Indicates adults meeting antihypertensive treatment eligibility under both 2017 and 2025 AHA/ACC guideline.

d Adjusted for NHANES survey weights to represent the national U.S. population of noninstitutionalized adults.

e Determined using the AHA Pooled Cohort Equation function.

### Newly eligible under 2025 guideline

Table 2 summarizes the characteristics of adults newly eligible for antihypertensive therapy under the 2025 AHA/ACC guideline. This group comprised approximately half a million U.S. adults and was predominantly female, older, and largely without established ASCVD. Newly eligible adults were primarily non-Hispanic White, with representation from non-Hispanic Black, Hispanic, and Asian groups. Despite a high insurance coverage, employment rates were lower and educational attainment was generally modest. Clinically, newly eligible adults exhibited elevated cardiometabolic risk, including obesity, stage 1 blood pressure levels, and mildly elevated glycemic markers, consistent with metabolic vulnerability rather than overt diabetes.

**Table 2:** Demographic Characteristics of the Newly Eligible Population Under the 2025 Guideline

Weighted sample size	522,699
Age, y (SD)	64.042 (3.051)
Sex	
Female	457,773 (87.6%)
Male	64,927 (12.4%)
Race/ethnicity	
Mexican American	15,349 (2.9%)
Non-Hispanic Asian	8,129 (1.6%)
Non-Hispanic Black	82,424 (15.8%)
Non-Hispanic White	403,673 (77.2%)
Other Hispanic	13,125 (2.5%)
Other/multiracial	15,349 (2.9%)
Education level	
College graduate or above	96,833 (18.5%)
High school graduate/GED or equivalent	220,372 (42.2%)
Less than 9th grade	66,580 (12.7%)
Some college or AA degree	138,915 (26.6%)
Marital status	
Married/living with partner	289,648 (55.4%)
Never married	21,237 (4.1%)
Widowed/divorced/separated	211,815 (40.5%)
Income	
Family income to poverty ratio	3.370 (1.401)
Biometrics	
BMI (kgm^2^)	32.861 (6.402)
SBP (mm Hg)	133.008 (5.453)
DBP (mm Hg)	76.168 (5.838)
Blood glucose (mgdL)	104.176 (6.116)
HbA1c	5.865 (0.305)
Currently employed	
No	356,161 (68.1%)
Yes	166,538 (31.9%)
Covered by health insurance	
No	30,199 (5.8%)
Yes	492,500 (94.2%)
Prevent equations	
PREVENT_BASE_10	8.251 (1.639)
PREVENT_wACR_10	19.774 (2.674)
PREVENT_wHbA1c_10	16.665 (3.228)
PREVENT_Full_10	31.835 (3.353)
PREVENT_BASE_30	42.021 (4.109)
PREVENT_Full_30	7.317 (1.386)
PCE_10 y^a^	8.730 (1.368)
Prior ASCVD	
No	522,699 (100.0%)

Values are N survey-weighted (weighted %). Adjusted for NHANES survey weights to represent the national U.S. population of noninstitutionalized adults.

Abbreviations as in Table 1.

a Determined using the AHA Pooled Cohort Equation function.

### Concordant and discordant eligibility

Most adults were ineligible under both guidelines (63.3%), whereas 36.3% were eligible under both, and fewer than 1% were discordant. Those consistently eligible were older with higher cardiometabolic risk profiles, whereas consistently ineligible adults were younger and healthier. Newly eligible individuals under the 2025 guideline were predominantly older women with higher BMI but lower diastolic blood pressure, suggesting that the updated PREVENT-based thresholds identify additional women at elevated cardiovascular risk who may benefit from earlier intervention Table 3.

**Table 3:** Characteristics of Groups According to Concordant and Discordant Recommendations From 2017 and 2025 AHA/ACC Thresholds for Initiation of Blood Pressure Treatment

	2017 and 2025 Concordant		2017 and 2025 Discordant	
	Ineligible in Both	Eligible in Both	Eligible in 2017 But Not 2025	Eligible in 2025 But Not 2017
Overall				
N survey-weighted (weighted %)	101,732,820 (63.3%)	58,347,898 (36.3%)	101,045 (0.1%)	522,699 (0.3%)
Age, y (SD)	50 (13)	56 (13)	55 (6)	64 (3)
Women, weighted %	51.4	48.4	12.9	87.6
BMI, kg/m^2^ (SD)	29.7 (7.2)	31.0 (7.0)	29.5 (3.5)	32.5 (7.5)
SBP, mm Hg (SD)	114 (10)	139 (19)	132 (42)	133 (5)
DBP, mm Hg (SD)	70 (8)	84 (10)	82 (4)	76 (6)
Total cholesterol, mg/dL (SD)	190.3 (39.4)	194.3 (42.6)	194.9 (41.6)	182.7 (30.6)
HDL, mg/dL (SD)	53.8 (15.8)	53.6 (16.5)	47.6 (16.2)	51.7 (11.1)
eGFR, mL/min per 1.73 m^2^ (SD)	94.5 (18.2)	90.5 (19.0)	91.8 (12.7)	79.7 (13.3)
Current smoker, weighted %	16.0	18.6	48.6	28.2
Diabetes, weighted %	51.1	73.9	0.0	0.0
PCE_10 y (SD)	6.7 (8.2)	15.9 (14.3)	11.6 (2.4)	7.3 (1.4)
PREVENT_BASE_10 (SD)	6.2 (6.5)	12.4 (8.7)	6.5 (1.1)	8.7 (1.4)
Aged <60 y				
N survey-weighted (weighted %)	73,553,238 (68.7)	33,360,359 (31.2)	88,024 (0.1)	26,361 (<0.1)
Age, y (SD)	44 (9)	47 (9)	53 (3)	57 (3)
Women, weighted %	51.1	46.2	<1.0	<1.0
BMI, kg/m^2^	29.7 (7.4)	32.1 (7.5)	29.4 (2.5)	48.5 (1.3)
SBP, mm Hg	114 (10)	136 (14)	132 (4)	127 (17)
DBP, mm Hg	71 (8)	87 (8)	82 (5)	84 (5)
Total cholesterol, mg/dL	191.5 (38.6)	197.4 (39.7)	197.8 (44.1)	142.6 (6.4)
HDL, mg/dL	53.3 (15.1)	52.2 (16.4)	43.5 (12.6)	40.5 (3.5)
eGFR, mL/min per 1.73 m^2^	100.1 (15.5)	98.1 (16.8)	95.0 (9.5)	87.6 (27.2)
Current smoker, weighted %	17.8	23.9	55.8	100.0
Diabetes, weighted %	48.1	75.2	0.0	0.0
PCE_10 y	3.2 (3.6)	7.8 (7.4)	11.8 (2.5)	7.9 (2.5)
PREVENT_BASE_10	3.4 (3.0)	7.7 (6.2)	6.4 (1.2)	7.7 (0.2)
Aged ≥60 y^a^				
N survey-weighted (weighted %)	28,179,583 (52.5)	24,987,539 (46.6)	-	496,338 (0.9)
Age, y (SD)	67 (5)	69 (6)	-	64 (3)
Women, weighted %	52.3	52.0	-	89.6
BMI, kg/m^2^	29.9 (6.5)	29.9 (6.4)	-	32.0 (5.4)
SBP, mm Hg	116 (9)	144 (14)	-	133 (5)
DBP, mm Hg	67 (7)	79 (10)	-	76 (6)
Total cholesterol, mg/dL	187.4 (41.3)	190.0 (45.8)	-	184.9 (29.9)
HDL, mg/dL	55.1 (17.6)	55.5 (16.6)	-	52.3 (11.1)
eGFR, mL/min per 1.73 m^2^	79.8 (16.6)	80.4 (16.9)	-	79.3 (12.8)
Current smoker, weighted %	11.3	11.5	-	24.4
Diabetes, weighted %	58.9	72.1	-	0.0
PCE_10 yr	15.7 (10.0)	26.6 (14.2)	-	7.3 (1.4)
PREVENT_BASE_10	13.7 (7.2)	18.7 (7.6)	-	8.8 (1.4)

eGFR = estimated glomerular filtration rate; HDL = high-density lipoprotein; other abbreviations as in Table 1.

a Sample not large enough to calculate survey-weighted population counts and proportions for those eligible in 2017 but not 2025.

### Projected differences in number and proportions of U.S. adults recommended for blood pressure treatment

The 2025 AHA/ACC hypertension guideline is projected to result in a minimal overall increase in the treatment eligibility compared with the 2017 guideline. Nationally, an estimated 420,000 more adults (0.7% increase) would be recommended for blood pressure treatment under the 2025 thresholds. This expansion is driven primarily by older adults (≥60 years), with a 1.9% increase (approximately 480,000 individuals), whereas no meaningful change is observed among those <60 years. By sex, eligibility rises among women (+1.6%) but remains unchanged among men. Across racial and ethnic groups, the largest relative increase occurs among non-Hispanic White adults (+1.1%), with minimal changes among other groups. These findings indicate that the new 2025 guideline—guided by the PREVENT risk framework—broadens treatment recommendations slightly, particularly among older adults and women, without substantially altering eligibility patterns across most demographic groups Table 4.

**Table 4:** Projected Differences in Number and Proportions of U.S. Adults Receiving or Recommended for Blood Pressure Treatment

	With 2017, in Millions	With 2025, in Millions	Difference (95% CI), in Hundred-Thousands	Difference (95% CI), %
Overall	58.4 (54.3-62.6)	58.9 (54.6-63.1)	4.2 (0.4-8.1)	0.7 (0.1-1.4)
Age				
Young (<60 y)	33.4 (30.7-36.2)	33.4 (30.6-36.2)	−0.6 (−1.3-0)	−0.2 (−0.4-0)
Older (≥ 60 y)	25 (21.8-28.2)	25.5 (22.3-28.7)	4.8 (1-8.7)	1.9 (0.4-3.5)
Sex				
Females	28.4 (24.8-32)	28.8 (25.2-32.5)	4.4 (0.6-8.2)	1.6 (0.2-2.9)
Males	30 (27.5-32.6)	30 (27.4-32.6)	−0.2 (−0.9-0.5)	−0.1 (−0.3-0.2)
Race/ethnicity				
Mexican American	3.6 (2.7-4.6)	3.7 (2.7-4.6)	0.2 (−0.1-0.4)	0.4 (−0.2-1)
Non-Hispanic Asian	3.2 (2.2-4.2)	3.2 (2.2-4.2)	−0.1 (−0.4-0.2)	−0.3 (−1.3-0.7)
Non-Hispanic Black	8.5 (6.3-10.7)	8.6 (6.4-10.7)	0.4 (−0.3-1.2)	0.5 (−0.4-1.4)
Non-Hispanic White	36.1 (30.8-41.5)	36.5 (31-42.1)	3.8 (0-7.6)	1.1 (0-2.1)
Other Hispanic	4.3 (3.2-5.5)	4.3 (3.2-5.5)	−0.1 (−0.6-0.4)	−0.2 (−1.3-0.9)
Other/multiracial	2.6 (1.8-3.5)	2.6 (1.8-3.5)	0 (0-0)	0 (0-0)

### Concordance and discordance in eligibility using different versions of the PREVENT model to estimate 2025 eligibility

**Figure 1:**
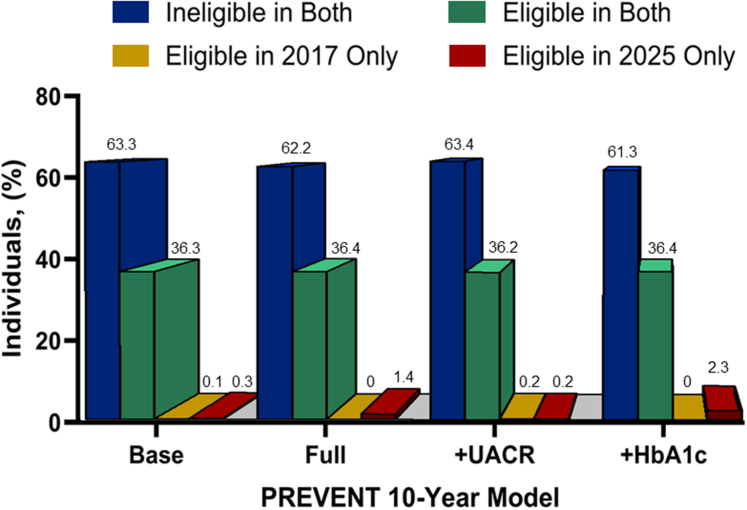
**Concordant and Discordant Hypertension Treatment Eligibility** This figure shows the proportion of U.S. adults classified as concordantly ineligible, concordantly eligible, newly eligible under the 2025 guideline, or no longer eligible under the 2025 guideline when comparing the 2017 and 2025 AHA/ACC hypertension guidelines. Results are based on simulated application of guideline criteria among NHANES participants aged 30 to 79 years included in the analytic sample (unweighted n = 5,578). Overall eligibility reflects adults meeting treatment thresholds under the 2025 guideline when applied to the full sample, whereas newly eligible adults represent those who met treatment thresholds under the 2025 guideline but not under the 2017 guideline. Estimates are shown separately for each PREVENT equation variant (Base, Full, albumin-to-creatinine ratio, and HbA1c). Across all models, most adults were concordantly ineligible, approximately one-third were concordantly eligible, and only a small proportion were newly eligible or no longer eligible. HbA1c = hemoglobin A1c; PREVENT = predicting risk of cardiovascular disease events.

Figure 1 illustrates the concordance and discordance in treatment eligibility between the 2017 and 2025 guidelines when applying different PREVENT equation versions to estimate 2025 eligibility. Across all models, most adults were concordantly ineligible (61%-63%) and roughly one-third were concordantly eligible (36%). Discordance was minimal, although more individuals became newly eligible under the 2025 guideline than lost eligibility. The PREVENT HbA1c model identified the largest proportion of new eligibles (23%), followed by the Full model (1.4%), whereas the Base and ACR models yielded smaller changes (<0.5%). Very few individuals were eligible under the 2017 but not 2025 guideline across all models (<0.2%). Overall, findings indicate that eligibility status remains largely consistent across models, but inclusion of glycemic markers in the HbA1c and Full versions slightly broadens treatment recommendations under the 2025 guideline (Supplemental Table 2).

### Predictors of eligibility using PREVENT base 10-year CVD risk

**Figure 2:**
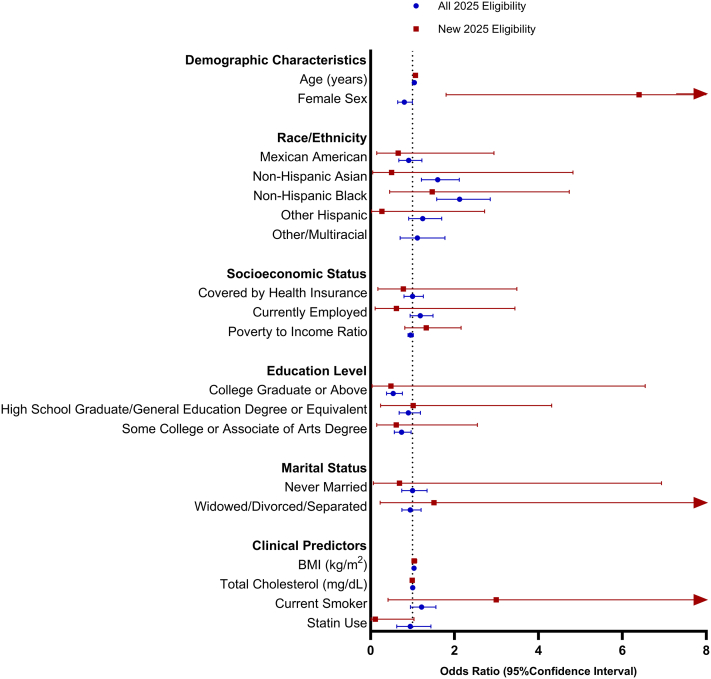
**Predictors of Hypertension Treatment Eligibility Under 2025 Guideline** This figure presents adjusted ORs (95% CIs) for demographic, socioeconomic, and clinical factors associated with meeting guideline-based treatment thresholds for antihypertensive therapy under the 2025 AHA/ACC hypertension guideline. Analyses were conducted using the PREVENT Base 10-year cardiovascular disease risk equation and include NHANES participants aged 30 to 79 years (unweighted n = 5,578). Eligibility reflects simulated classification based on guideline criteria rather than observed treatment initiation or medication use. Multivariable logistic regression models were adjusted for age, sex, race and ethnicity, educational attainment, employment status, insurance status, marital status, body mass index, diabetes, and chronic kidney disease. Survey weights were applied to generate nationally representative estimates. BMI = body mass index.

In multivariate analysis, eligibility based on the 2025 AHA/ACC guidelines, using the PREVENT Base 10-year CVD risk model, was associated with older age, higher BMI, and non-Hispanic Asian and non-Hispanic Black race or ethnicity, each of which were associated with higher odds of treatment eligibility (Figure 2). In contrast, having some college education or college degree or higher was associated with lower odds. Newly eligible adults were more often females, with sex emerging as the primary distinguishing predictor in this group. Other demographic, socioeconomic, and clinical characteristics showed minimal variation across eligibility categories.

### Predictors of eligibility using PREVENT Full 10-year CVD risk

Multivariate analysis revealed that using the PREVENT Full 10-year CVD risk model, predictors of eligibility remained consistent with those observed in the Base model, with older age, higher BMI, and non-Hispanic Asian and non-Hispanic Black adults more likely to meet treatment criteria whereas those with a college degree or greater had lower odds (Supplemental Figure 2). Female sex continued to distinguish newly eligible adults, with a higher magnitude of association in this model compared with the Base equation. Current smoking also emerged as an additional predictor in the Full model, associated with both overall and new eligibility. Educational attainment, socioeconomic indicators, and other demographic factors showed limited variation.

### Predictors of eligibility using PREVENT ACR 10-year CVD risk

Using the PREVENT 10-year CVD risk model incorporating the ACR, predictors of overall eligibility were similar to those in the Base and Full models, with older age, higher BMI, and non-Hispanic Asian and non-Hispanic Black adults showing higher odds of eligibility (Supplemental Figure 3). Educational attainment also remained a consistent factor, with adults holding a college degree or higher, as well as those with some college education, less likely to be eligible compared with those without a high school diploma. Sex was not a distinguishing predictor in this model, and no clear differences were observed among newly eligible adults.

### Predictors of eligibility using PREVENT HbA1c 10-year CVD risk

Using the PREVENT 10-year CVD risk model incorporating HbA1c, predictors of overall eligibility closely aligned with prior models, with older age, higher BMI, and non-Hispanic Asian and non-Hispanic Black adults showing higher odds of treatment eligibility (Supplemental Figure 4). Employment status emerged as a new predictor in this model, with currently employed adults more likely to be eligible, and this association was stronger among those newly eligible under the 2025 guideline. Educational attainment continued to differentiate eligibility, with adults holding a college degree or higher and those with some college or an associate’s degree less likely to be eligible. Sex was not a significant distinguishing factor in this model.

Supplemental Table 3 compares adults meeting eligibility criteria for antihypertensive therapy under the 2025 AHA/ACC guideline across four PREVENT equation variants. Overall eligibility was similar across models, with modest variation in prevalence. Demographic and socioeconomic characteristics were largely consistent across models, including age, sex, race and ethnicity, education, employment, and insurance status. Clinical profiles, including blood pressure and glycemic measures, were also comparable, with only minor differences in estimated cardiovascular risk across PREVENT variants.

## Discussion

In this nationally representative study, the 2025 AHA/ACC hypertension guideline produced a minimal overall expansion in treatment eligibility compared with the 2017 guidelines. Although early modeling suggested that adopting the PREVENT equations could lower treatment eligibility^21^ if risk thresholds were simply ported over, our findings indicate a slight increase.^2^^,^^22^, ^23^, ^24^ However, although we see an increase in eligibility, this expansion was not uniform across the population. The most notable gains occurred among older women with obesity and borderline glycemic abnormalities who are at an elevated long-term cardiovascular risk. This pattern aligns with PREVENT’s model design, which estimates total CVD (including heart failure), extends the age range down to 30 years, and anchors stage 1 treatment at a 7.5% 10-year CVD risk threshold.^8^^,^^25^

Cardiometabolic factors continued to play a defining role in treatment eligibility. Adults classified as eligible under either guidelines exhibited higher BMI, blood pressure, fasting glucose, and HbA1c levels than those ineligible, consistent with the strong link between metabolic health and cardiovascular risk. However, the newly eligible population under the 2025 guideline was characterized less by overt diabetes or chronic kidney disease and more by subclinical metabolic vulnerability, suggesting that PREVENT’s inclusion of broader cardiometabolic inputs may allow for finer detection of cumulative risk in primary prevention.^26^, ^27^, ^28^

In parallel, sociodemographic factors contributed more modestly but remain important. Eligibility increased slightly among women but remained stable among men. Patterns by race and ethnicity were largely unchanged, with non-Hispanic Black adults continuing to represent a disproportionately high share of treatment-eligible individuals, whereas non-Hispanic White adults accounted for most of the absolute increase in eligibility. Educational attainment showed inverse associations, consistent with the established gradient linking lower education and higher cardiovascular risk. Importantly, PREVENT is race-agnostic, a deliberate design choice to avoid encoding a social construct as biology while maintaining or improving calibration and discrimination across racial and ethnic groups compared with the PCEs.^29^^,^^30^ Our observed pattern, an inclusive yet minimal expansion concentrated among metabolically at-risk adults, aligns with PREVENT’s intent to improve equity in the cardiovascular risk assessment. Continued attention to equitable implementation through electronic health record –based automation and shared decision-making will be essential to ensure these refinements translate into better control and outcomes.^25^

Although the overall increase in eligibility was minimal, notable differences emerged between PREVENT equation variants. Across all models, the overarching pattern was consistent: most adults remained either consistently eligible or consistently ineligible under both the 2017 and 2025 guidelines, and only a small proportion changed status. Yet the magnitude and composition of newly eligible adults varied by model specification. The Base model produced results most aligned with 2017 eligibility, highlighting younger adults with diabetes and reflecting minimal expansion. The Full model, which incorporates additional predictors such as BMI, HbA1c, and kidney function, modestly increased eligibility (1.4%) by identifying adults with elevated metabolic or renal risk. The ACR model generated patterns nearly identical to the Base model, suggesting that albuminuria contributes little incremental value at the population level. In contrast, the HbA1c model expanded eligibility most substantially (2.3%), primarily capturing older adults with modest glycemic elevations but without overt diabetes or established CVD.

The consistency observed across the PREVENT equation variants suggests that the observed expansion is not simply a feature of one model but a robust finding across multiple approaches, further reinforcing the reliability of PREVENT as a contemporary framework for the population-level cardiovascular risk assessment. At the same time, PREVENT’s modular design^7^^,^^8^ which allows integration of markers such as HbA1c or ACR^31^^,^^32^ provides an opportunity for more precise risk stratification in settings where these data are available, further enhancing its value as a foundation for risk-based blood pressure management. These variations also have practical implications for implementation. The Base and ACR models may serve as pragmatic tools for population surveillance or for primary care environments where laboratory data are limited, whereas the Full and HbA1c models may enhance precision within integrated health systems that can leverage richer electronic health record data. Incorporating glycemic measures appears to capture a broader spectrum of cardiometabolic risk, particularly among older women with obesity and modest HbA1c elevations, reinforcing PREVENT’s design as a flexible, scalable framework adaptable to real-world practice settings.^25^ These findings also help to contextualize recent NHANES-based analyses that have reported substantially larger projected expansions in antihypertensive medication recommendations under the 2025 guideline. Such estimates primarily reflect inclusion of adults with stage 1 hypertension and low predicted CVD risk who may be considered for pharmacologic therapy only if blood pressure remains elevated after a trial of lifestyle intervention. By contrast, our analysis focused on guideline-based treatment eligibility at the time of assessment and adhered to the validated PREVENT age range, yielding estimates that more directly reflect changes in treatment thresholds rather than conditional, future-oriented care pathways.^33^

International guideline comparisons further contextualize how these U.S. changes may play out in practice. The 2025 U S. guideline maintains a stage 1 threshold of ≥130/80 mm Hg and a uniform treatment target <130/<80, whereas the European Society of Cardiology 2024 and European Society of Hypertension 2023 define hypertension at ≥140/90 mm Hg and adopt more conservative initiation thresholds and age-stratified targets.^34^, ^35^, ^36^ As a result, many U.S. “stage 1” adults, in our newly eligible group, particularly older women with metabolic risk, would be managed initially with lifestyle alone under European frameworks unless very high risk is present. These divergent thresholds and targets underscore that downstream effects on medication initiation and control rates will vary by health-system philosophy, even when the underlying risk phenotype is the same.^37^

Taken together, our analysis operationalizes the AHA/ACC Scientific Statement’s risk-based framework at the population level. By adopting PREVENT, broadening outcomes to total CVD, and anchoring treatment at a 7.5% 10-year risk, the 2025 guideline refines rather than redefines eligibility for antihypertensive therapy. This refinement minimally shifts treatment earlier in the life course, particularly toward older women with obesity and metabolic vulnerability, who face elevated lifetime risk even in the absence of established disease. The challenge ahead lies not in identifying large numbers of new patients but in ensuring that these targeted refinements translate into improved treatment and outcomes.

### Study Limitations

This study should be interpreted considering several limitations. First, the cross-sectional design precludes causal inference about whether expanded eligibility will translate into improved outcomes. Second, the PREVENT models were applied using available NHANES data, and although they are validated, measurement error and residual confounding are possible. This also includes the limitation that some data are not provided in the publicly available NHANES data set, specifically the social deprivation index. Third, some subgroup estimates had limited precision, particularly when stratified by race and ethnicity, because of small sample sizes and survey design constraints. Finally, all our analysis quantifies eligibility shifts; it does not capture treatment uptake, adherence, or real-world effectiveness, which will determine the ultimate impact of the guidelines on cardiovascular health.

## Conclusions

The adoption of the 2025 AHA/ACC hypertension guideline represents an important refinement in risk-based treatment assessment through incorporation of the PREVENT equations. Using nationally representative data, we found that the updated guideline results in a minimal expansion in antihypertensive treatment eligibility, driven primarily by older women with metabolic vulnerability, with largely stable classification across PREVENT model variants. These findings indicate that the 2025 guideline fine-tunes rather than fundamentally alters treatment thresholds, minimally extending preventive consideration to adults at elevated cardiovascular risk without established disease.

## Funding support and author disclosures

The authors have reported that they have no relationships relevant to the contents of this paper to disclose.
