# Supplementary material for: Shifting Thresholds: Changes in Antihypertensive Eligibility Under the 2025 Versus 2017 Hypertension Guidelines
Source: JACC Adv. 2026 Jan 21;5(2):102546. doi: 10.1016/j.jacadv.2025.102546 (PMC12859489; doi:10.1016/j.jacadv.2025.102546)
Supplement: Supplementary Figures 1 to 4 and Supplementary Tables 1 to 7 [file mmc1.docx]

**Title**: Shifting Thresholds: Changes in Antihypertensive Eligibility Under the 2025 Versus 2017 Hypertension Guidelines

**Authors:**

Faith E. Metlock^1^; Bede N. Nriagu^1^; Britton Scheuermann^2^; Carl Ade^2^; Yaa Adoma Kwapong^3^; Alexander C. Razavi^4^; Stephen Juraschek^5^; Sharmaine M. McCoy^1^; Lily N. Dastmalchi^1^, Garima Sharma^1^, Jared A. Spitz^1^,

^1^Inova Schar Heart and Vascular, Inova Health System, Falls Church, VA

^2^College of Health and Human Sciences, Kansas State University, Manhattan

^3^Johns Hopkins School of Medicine, Baltimore, MD

^4^Emory Clinical Cardiovascular Research Institute, Emory University School of Medicine, Atlanta, GA,

^5^Beth Israel Deaconess Medical Center, Harvard Medical School, Boston, MA

**Corresponding Author:**

Jared A. Spitz

Inova Health System

8081 Innovation Drive

Falls Church, VA
[jared.spitz@inova.org](mailto:jared.spitz@inova.org)

**Supplement Document**


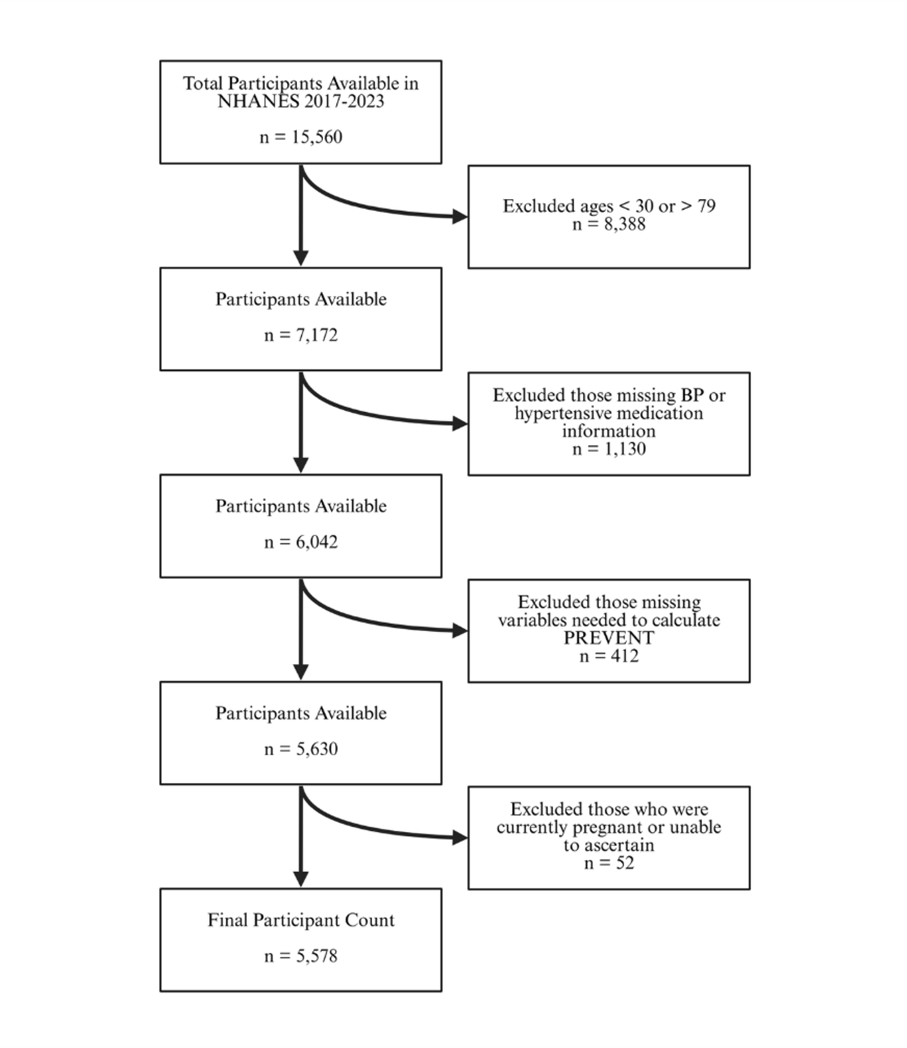


NHANES 2017-2020

**Supplemental Figure 1. Cohort Determination Flowchart for Blood Pressure Guidelines Analysis.** Flow diagram illustrating the selection of the analytic sample from the National Health and Nutrition Examination Survey (NHANES) 2017–2020 cycles. Adults aged 30–79 years were included if they had complete data for blood pressure, antihypertensive medication use, and covariates required to estimate eligibility under the 2017 ACC/AHA and 2025 AHA/ACC/ hypertension guidelines. Participants with missing data on key variables or a history of cardiovascular disease were excluded. Final analytic sample sizes are shown at each step.





**Supplemental Figure 2. Predictors of Eligibility for Antihypertensive Therapy Using the PREVENT Full 10-Year CVD Risk Model.** Adjusted odds ratios (95% CI) are shown for demographic, socioeconomic, and clinical predictors of eligibility under the 2025 AHA/ACC blood pressure guideline, using the PREVENT Full 10-year risk equation, which incorporates additional variables such as HbA1c and albuminuria when available. Models were adjusted for age, sex, race/ethnicity, education, employment, insurance, marital status, BMI, diabetes, and CKD.


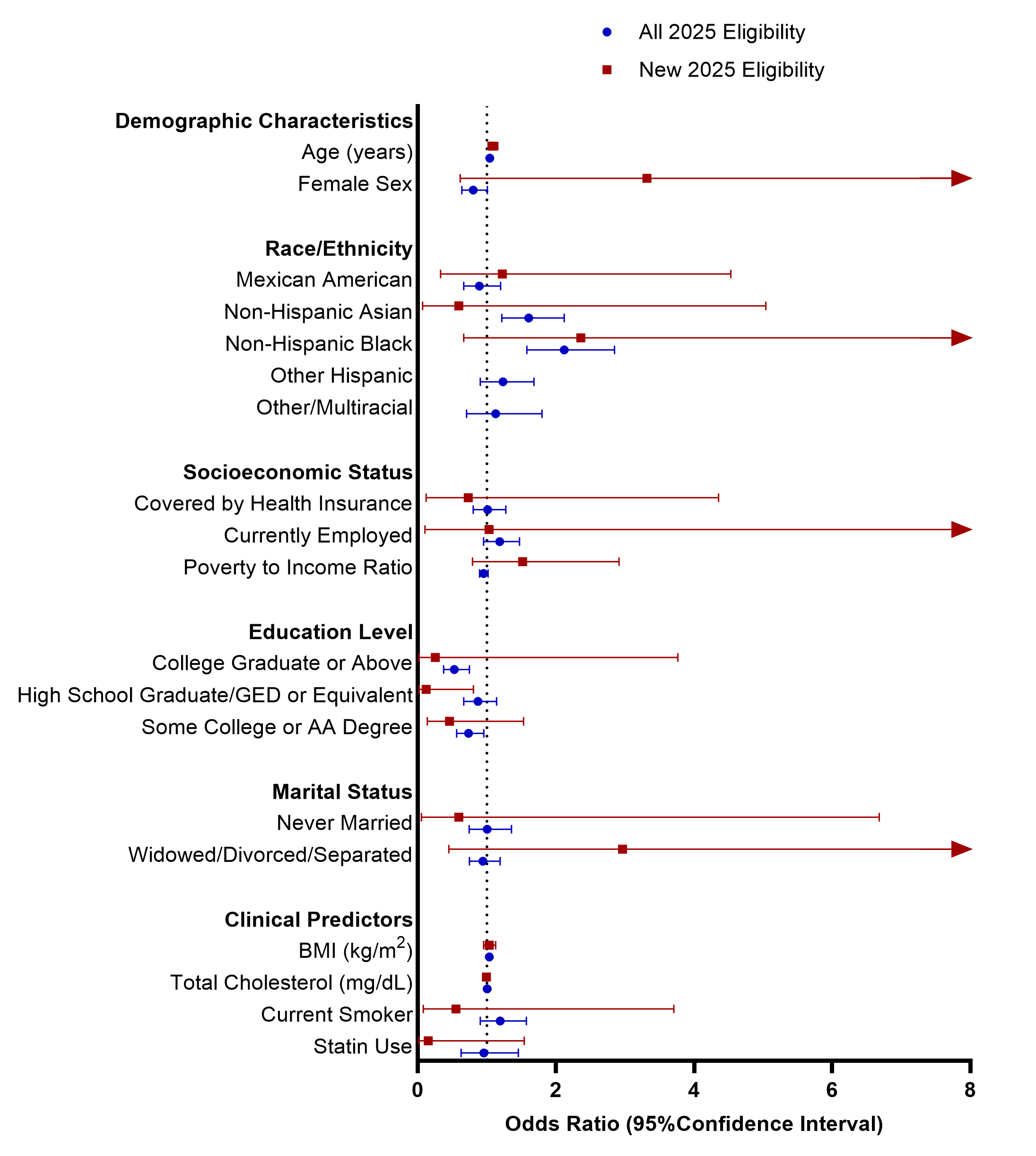


**Supplemental Figure 3. Predictors of Eligibility for Antihypertensive Therapy Using the PREVENT ACR 10-Year CVD Risk Model.** Forest plot showing multivariable-adjusted odds ratios (ORs) and 95% confidence intervals (CIs) for predictors of overall (blue) and new (pink) eligibility for antihypertensive therapy among U.S. adults aged 30–79 years in NHANES 2017–2020. Eligibility was determined using the 2025 AHA/ACC hypertension guideline incorporating the PREVENT ACR (albumin-to-creatinine ratio) 10-year cardiovascular disease (CVD) risk model. Predictors are grouped by demographic, race/ethnicity, socioeconomic, educational, marital, and clinical factors. Results highlight the sociodemographic and clinical characteristics associated with both overall and newly identified treatment eligibility under the updated guideline.





**Figure 4. Predictors of Eligibility for Antihypertensive Therapy Using the PREVENT HbA1C 10-Year CVD Risk Model.** Forest plot showing multivariable-adjusted odds ratios (ORs) and 95% confidence intervals (CIs) for predictors of overall (blue) and new (pink) eligibility for antihypertensive therapy among U.S. adults aged 30–79 years in NHANES 2017–2020. Eligibility was defined using the 2025 AHA/ACC hypertension guideline incorporating the PREVENT HbA1C 10-year cardiovascular disease (CVD) risk model. Predictors are categorized by demographic, socioeconomic, educational, marital, and clinical characteristics. Results illustrate differences in factors associated with individuals newly qualifying for treatment under the updated guideline compared with prior criteria

| **Supplementary Table 1. BP levels used to define antihypertensive treatment thresholds according to the 2017 and 2025 ACC/AHA guidelines.** | | |
| --- | --- | --- |
|  | **2017 ACC/AHA** | **2025 AHA/ACC** |
| Non-existing clinical CVD (coronary heart disease, stroke, heart failure) | systolic BP ≥140 or diastolic BP ≥90 mm Hg | systolic BP ≥140 or diastolic BP ≥90 mm Hg |
| Existing clinical CVD or significant risk factor (coronary heart disease, stroke, heart failure, diabetes, chronic kidney disease) | systolic BP 130 to <140 mm Hg and diastolic <90 or diastolic BP 80 to <90 mm Hg and systolic BP <140 | systolic BP 130 to <140 mm Hg and diastolic <90 or diastolic BP 80 to <90 mm Hg and systolic BP <140 |
| High CVD risk | systolic BP 130 to <140 mm Hg and diastolic <90 or diastolic BP 80 to <90 mm Hg and systolic BP <140 with 10-year PCE risk ≥10% | systolic BP 130 to <140 mm Hg and diastolic <90 or diastolic BP 80 to <90 mm Hg and systolic BP <140 with 10-year total CVD risk ≥7.5% with PREVENT-CVD |
| ACC/AHA = American College of Cardiology/American Heart Association, BP = blood pressure, CVD = cardiovascular disease, PCE = Pooled-Cohort Equation | | |

| **Supplemental Table 2. Concordance and Discordance in Eligibility for Blood Pressure Treatment Based on 2017 vs. 2025 guidelines, Using Different Versions of the PREVENT Model to Estimate 2025 Eligibility** | | | | |
| --- | --- | --- | --- | --- |
|  | **2017 and 2025 Concordant** | | **2017 and 2025 Discordant** | |
|  | **Ineligible in both** | **Eligible in both** | **Eligible in 2017 but not 2025** | **Eligible in 2025 but not 2017** |
| Using the PREVENT Base Model | 101,732,820 (63.3%) | 58,347,898 (36.3%) | 101,045 (0.1%) | 522,699  (0.3%) |
| Using the PREVENT Full Model | 99,949,168 (62.2%) | 58,441,814 (36.4%) | 7,129  (0.0%) | 2,306,351 (1.4%) |
| Using the PREVENT ACr Model | 101,931,556 (63.4%) | 58,182,402 (36.2%) | 266,541 (0.2%) | 323,963  (0.2%) |
| Using the PREVENT HBA1C Model | 98,590,054 (61.3%) | 58,448,943 (36.4%) | 0  (0.0%) | 3,665,466 (2.3%) |

| **Supplemental Table 3: Baseline characteristics among individuals eligible under 2017 and 2025 guidelines using the various PREVENT Equation models** | | | | | |
| --- | --- | --- | --- | --- | --- |
|  | **Eligible under 2017 guideline** | **Eligible under 2025 guideline (PREVENT base model)** | **Eligible under 2025 guideline (PREVENT full model)** | **Eligible under 2025 guideline (PREVENT ACr model)** | **Eligible under 2025 guideline (PREVENT HBA1C model)** |
| N Survey-weighted (weighted %) | 58,448,943 | 58,870,597 | 60,748,165 | 58,506,365 | 62,114,409 |
| Age, Yrs (SD) | 56.327 (12.858) | 56.398  (12.834) | 56.468  (12.661) | 56.369  (12.865) | 56.367  (12.551) |
| Sex | | | | | |
| Female | 28,402,313 (48.6%) | 28,847,065 (49.0%) | 30,084,632 (49.5%) | 28,652,245 (49.0%) | 30,369,071 (48.9%) |
| Male | 30,046,630 (51.4%) | 30,023,532 (51.0%) | 30,663,533 (50.5%) | 29,854,120 (51.0%) | 31,745,338 (51.1%) |
| Race Ethnicity | | | | | |
| Mexican American | 3,641,412 (6.2%) | 3,656,760 (6.2%) | 3,822,219  (6.3%) | 3,609,713 (6.2%) | 3,946,356  (6.4%) |
| Non-Hispanic Asian | 3,224,697 (5.5%) | 3,214,983 (5.5%) | 3,364,008  (5.5%) | 3,214,983 (5.5%) | 3,415,876  (5.5%) |
| Non-Hispanic Black | 8,510,702 (14.6%) | 8,552,053 (14.5%) | 8,712,960 (14.3%) | 8,509,182 (14.5%) | 8,820,823 (14.2%) |
| Non-Hispanic White | 36,125,859 (61.8%) | 36,508,484 (62.0%) | 37,731,600 (62.1%) | 36,263,700 (62.0%) | 38,705,727 (62.3%) |
| Other Hispanic | 4,326,721 (7.4%) | 4,318,764 (7.3%) | 4,409,272  (7.3%) | 4,289,234 (7.3%) | 4,501,001  (7.2%) |
| Other/Multiracial | 2,619,553 (4.5%) | 2,619,553 (4.4%) | 2,708,106  (4.5%) | 2,619,553 (4.5%) | 2,724,627  (4.4%) |
| Education Level | | | | | |
| College Graduate or Above | 14,875,760 (25.5%) | 14,941,730 (25.4%) | 15,343,724 (25.3%) | 14,851,547 (25.4%) | 15,830,977 (25.5%) |
| High School Graduate/GED or Equivalent | 17,322,875 (29.6%) | 17,525,837 (29.8%) | 18,108,876 (29.8%) | 17,270,841 (29.5%) | 18,473,565 (29.8%) |
| Less than High School | 7,640,410 (13.1%) | 7,679,721 (13.1%) | 7,811,606 (12.9%) | 7,679,721 (13.1%) | 8,114,378 (13.1%) |
| Some College or AA Degree | 18,587,093 (31.8%) | 18,700,505 (31.8%) | 19,461,154 (32.0%) | 18,681,451 (31.9%) | 19,672,684 (31.7%) |
| Marital Status | | | | | |
| Married/Living With Partner | 39,080,119 (66.9%) | 39,286,196 (66.8%) | 40,278,936 (66.3%) | 39,057,008 (66.8%) | 41,386,638 (66.6%) |
| Never Married | 5,793,972 (9.9%) | 5,815,209 (9.9%) | 6,010,765  (9.9%) | 5,802,175 (9.9%) | 6,028,383  (9.7%) |
| Widowed/Divorced/Separated | 13,556,497 (23.2%) | 13,750,836 (23.4%) | 14,440,108 (23.8%) | 13,628,826 (23.3%) | 14,681,032 (23.6%) |
| Currently Employed | | | | | |
| No | 23,818,951 (40.8%) | 24,133,097 (41.0%) | 24,673,977 (40.7%) | 23,941,639 (41.0%) | 24,932,136 (40.2%) |
| Yes | 34,577,800 (59.2%) | 34,685,308 (59.0%) | 36,021,996 (59.3%) | 34,512,534 (59.0%) | 37,130,081 (59.8%) |
| Health Insurance Coverage | | | | | |
| No | 6,418,139 (11.0%) | 6,427,290 (10.9%) | 6,557,888 (10.8%) | 6,366,915 (10.9%) | 6,784,177 (10.9%) |
| Yes | 52,001,783 (89.0%) | 52,414,286 (89.1%) | 54,161,256 (89.2%) | 52,110,429 (89.1%) | 55,301,211 (89.1%) |
| Prevent Equations | | | | | |
| PREVENT_BASE_10 score | 12.379  (8.738) | 12.357  (8.711) | 12.153  (8.652) | 12.383  (8.731) | 11.972  (8.642) |
| PREVENT_wACR_10 score (Mean, SD) | 11.857  (9.313) | 11.836  (9.283) | 11.627  (9.213) | 11.866  (9.304) | 11.442  (9.193) |
| PREVENT_wHbA1c_10 score (Mean, SD) | 20.051 (13.064) | 20.059  (13.016) | 19.845  (12.874) | 20.068  (13.056) | 19.609  (12.830) |
| PREVENT_Full_10 score (Mean, SD) | 18.068 (13.198) | 18.070  (13.149) | 17.818  (13.021) | 18.098  (13.184) | 17.558  (12.992) |
| PREVENT_BASE_30 score (Mean, SD) | 33.714 (11.005) | 33.706  (10.968) | 33.468  (10.890) | 33.720  (10.998) | 33.207  (10.915) |
| PREVENT_Full_30 score (Mean, SD) | 38.593 (12.407) | 38.633  (12.369) | 38.472  (12.221) | 38.630  (12.407) | 38.209  (12.216) |
| PCE_10yr score (Mean, SD) | 15.844 (14.302) | 15.776  (14.272) | 15.490  (14.147) | 15.816  (14.305) | 15.259  (14.078) |
| Prior ASCVD | | | | | |
| No | 49,413,637 (84.5%) | 49,842,421 (84.7%) | 51,719,988 (85.1%) | 49,478,188 (84.6%) | 53,079,103 (85.5%) |
| Yes | 9,035,306 (15.5%) | 9,028,177 (15.3%) | 9,028,177 (14.9%) | 9,028,177 (15.4%) | 9,035,306 (14.5%) |

| **Supplemental Table 3. Concordance and Discordance in Eligibility for Blood Pressure Treatment Based on 2017 vs. 2025 guidelines, Using Different Versions of the PREVENT Model to Estimate 2025 Eligibility** | | | | |
| --- | --- | --- | --- | --- |
|  | **2017 and 2025 Concordant** | | **2017 and 2025 Discordant** | |
|  | **Ineligible in both** | **Eligible in both** | **Eligible in 2017 but not 2025** | **Eligible in 2025 but not 2017** |
| Using the PREVENT Base Model | 101,732,820 (63.3%) | 58,347,898 (36.3%) | 101,045 (0.1%) | 522,699  (0.3%) |
| Using the PREVENT Full Model | 99,949,168 (62.2%) | 58,441,814 (36.4%) | 7,129  (0.0%) | 2,306,351 (1.4%) |
| Using the PREVENT ACr Model | 101,931,556 (63.4%) | 58,182,402 (36.2%) | 266,541 (0.2%) | 323,963  (0.2%) |
| Using the PREVENT HBA1C Model | 98,590,054 (61.3%) | 58,448,943 (36.4%) | 0  (0.0%) | 3,665,466 (2.3%) |

| **Supplemental Table 4. Adjusted Odds Ratios (95% CI) for Predictors of Eligibility for Antihypertensive Therapy Using the PREVENT Base 10-Year CVD Risk Model** | | |
| --- | --- | --- |
|  | **All 2025 Eligible** | **New 2025 Eligible** |
| Demographics |  |  |
| Age (per year) | 1.04 (1.03-1.05) | 1.07 (1.03-1.12) |
| Female Sex | 1.24 (1.00-1.55) | 0.16 (0.04-0.56) |
| Race/Ethnicity |  |  |
| Mexican American | 0.91 (0.68-1.22) | 0.66 (0.15-2.94) |
| Non-Hispanic Asian | 1.60 (1.21-2.11) | 0.50 (0.05-4.82) |
| Non-Hispanic Black | 2.12 (1.58-2.85) | 1.47 (0.46-4.74) |
| Other Hispanic | 1.24 (0.91-1.70) | 0.27 (0.03-2.72) |
| Other/Multiracial | 1.12 (0.70-1.77) | - |
| Socioeconomic Factors |  |  |
| Covered by Health Insurance | 1.00 (0.80-1.26) | 0.78 (0.18-3.49) |
| Currently Employed | 1.18 (0.94-1.49) | 0.62 (0.11-3.44) |
| Poverty to Income Ratio | 0.95 (0.89-1.02) | 1.33 (0.82-2.16) |
| Education Level |  |  |
| College Graduate or Above | 0.54 (0.38-0.76) | 0.49 (0.04-6.54) |
| High School Graduate/GED or Equivalent | 0.90 (0.68-1.16) | 1.02 (0.24-4.32) |
| Some College or AA Degree | 0.74 (0.57-0.96) | 0.61 (0.15-2.55) |
| Marital Status |  |  |
| Never Married | 1.00 (0.74-1.35) | 0.69 (0.07-6.93) |
| Widowed/Divorced/Separated | 0.95 (0.75-1.20) | 1.51 (0.23-10.02) |
| Clinical Predictors |  |  |
| BMI (kg/m^2^) | 1.04 (1.02-1.05) | 1.04 (0.98-1.10) |
| Total Cholesterol (mg/dL) | 1.00 (1.00-1.01) | 0.99 (0.98-1.10) |
| Current Smoker | 1.22 (0.95-1.56) | 2.99 (0.42-21.54) |
| Statin Use | 0.95 (0.62-1.44) | 0.11 (0.01-1.03) |
| Models were adjusted for age, sex, race/ethnicity, education, employment, insurance, marital status, BMI, diabetes, and CKD | | |

| **Supplemental Table 5. Adjusted Odds Ratios (95% CI) for Predictors of Eligibility for Antihypertensive Therapy Using the PREVENT Full 10-Year CVD Risk Model** | | |
| --- | --- | --- |
|  | **All 2025 Eligible** | **New 2025 Eligible** |
| Demographics |  |  |
| Age (per year) | 1.05 (1.04-1.06) | 1.06 (1.05-1.08) |
| Female Sex | 0.83 (0.67-1.03) | 2.38 (1.22-4.62) |
| Race/Ethnicity |  |  |
| Mexican American | 0.94 (0.71-1.26) | 1.64 (0.54-5.01) |
| Non-Hispanic Asian | 1.62 (1.21-2.17) | 1.15 (0.40-3.28) |
| Non-Hispanic Black | 2.08 (1.56-2.77) | 0.67 (0.33-1.37) |
| Other Hispanic | 1.24 (0.90-1.69) | 0.67 (0.22-2.10) |
| Other/Multiracial | 1.12 (0.70-1.78) | 0.86 (0.09-8.03) |
| Socioeconomic Factors |  |  |
| Covered by Health Insurance | 1.04 (0.82-1.30) | 1.97 (0.66-5.87) |
| Currently Employed | 1.26 (0.99-1.60) | 2.09 (0.88-4.95) |
| Poverty to Income Ratio | 0.95 (0.88-1.02) | 0.98 (0.75-1.30) |
| Education Level |  |  |
| College Graduate or Above | 0.55 (0.40-0.76) | 1.18 (0.22-6.35) |
| High School Graduate/GED or Equivalent | 0.94 (0.70-1.25) | 2.01 (0.61-6.57) |
| Some College or AA Degree | 0.78 (0.60-1.01) | 1.96 (0.76-5.04) |
| Marital Status |  |  |
| Never Married | 1.03 (0.76-1.39) | 1.73 (0.91-3.28) |
| Widowed/Divorced/Separated | 0.98 (0.78-1.24) | 1.64 (0.71-3.79) |
| Clinical Predictors |  |  |
| BMI (kg/m^2^) | 1.03 (1.02-1.05) | 1.00 (0.96-1.04) |
| Total Cholesterol (mg/dL) | 1.00 (1.00-1.01) | 1.00 (1.00-1.01) |
| Current Smoker | 1.29 (1.02-1.63) | 2.67 (1.15-6.18) |
| Statin Use | 0.94 (0.64-1.40) | 0.66 (0.12-3.74) |
| Models were adjusted for age, sex, race/ethnicity, education, employment, insurance, marital status, BMI, diabetes, and CKD | | |

| **Supplemental Table 6. Adjusted Odds Ratios (95% CI) for Predictors of Eligibility for Antihypertensive Therapy Using the PREVENT+ARC 10-Year CVD Risk Model** | | |
| --- | --- | --- |
|  | **All 2025 Eligible** | **New 2025 Eligible** |
| Demographics |  |  |
| Age (per year) | 1.04 (1.03-1.05) | 1.09 (1.02-1.16) |
| Female Sex | 0.80 (0.64-1.01) | 3.32 (0.62-17.90) |
| Race/Ethnicity |  |  |
| Mexican American | 0.89 (0.66-1.20) | 1.22 (0.33-4.53) |
| Non-Hispanic Asian | 1.61 (1.22-2.12) | 0.59 (0.07-5.04) |
| Non-Hispanic Black | 2.12 (0.90-1.68) | 2.36 (0.66-8.36) |
| Other Hispanic | 1.23 (0.90-1.68) | - |
| Other/Multiracial | 1.13 (0.71-1.80) | - |
| Socioeconomic Factors |  |  |
| Covered by Health Insurance | 1.01 (0.80-1.27) | 0.73 (0.12-4.35) |
| Currently Employed | 1.19 (0.95-1.47) | 1.03 (0.10-10.31) |
| Poverty to Income Ratio | 0.95 (0.89-1.02) | 1.52 (0.79-2.94 |
| Education Level |  |  |
| College Graduate or Above | 0.53 (0.37-0.75) | 0.26 (0.02-3.76) |
| High School Graduate/GED or Equivalent | 0.87 (0.67-1.14) | 0.12 (0.02-0.81) |
| Some College or AA Degree | 0.74 (0.56-0.96) | 0.46 (0.14-1.53) |
| Marital Status |  |  |
| Never Married | 1.00 (0.74-1.36) | 0.59 (0.05-6.68) |
| Widowed/Divorced/Separated | 0.94 (0.75-1.19) | 2.96 (0.45-19.56) |
| Clinical Predictors |  |  |
| BMI (kg/m^2^) | 1.04 (1.02-1.05) | 1.04 (0.95-1.13) |
| Total Cholesterol (mg/dL) | 1.00 (1.00-1.01) | 0.99 (0.98-1.01) |
| Current Smoker | 1.19 (0.91-1.57) | 0.55 (0.08-3.71) |
| Statin Use | 0.95 (0.63-1.45) | 0.15 (0.02-1.54) |
| Models were adjusted for age, sex, race/ethnicity, education, employment, insurance, marital status, BMI, diabetes, and CKD | | |

| **Supplemental Table 7. Adjusted Odds Ratios (95% CI) for Predictors of Eligibility for Antihypertensive Therapy Using the PREVENT+HbA1c 10-Year CVD Risk Model** | | |
| --- | --- | --- |
|  | **All 2025 Eligible** | **New 2025 Eligible** |
| Demographics |  |  |
| Age (per year) | 1.05 (1.04-1.06) | 1.04 (1.03-1.06) |
| Female Sex | 0.80 (0.64-1.00) | 1.10 (0.71-1.71) |
| Race/Ethnicity |  |  |
| Mexican American | 0.92 (0.71-1.20) | 1.12 (0.42-3.02) |
| Non-Hispanic Asian | 1.58 (1.19-2.10) | 0.84 (0.48-1.47) |
| Non-Hispanic Black | 2.07 (1.55-2.77) | 0.76 (0.40-1.43) |
| Other Hispanic | 1.22 (0.90-1.65) | 0.67 (0.34-1.31) |
| Other/Multiracial | 1.09 (0.69-1.71) | 0.64 (0.10-4.26) |
| Socioeconomic Factors |  |  |
| Covered by Health Insurance | 1.03 (0.82-1.29) | 1.33 (0.41-4.33) |
| Currently Employed | 1.29 (1.03-1.61) | 2.20 (1.37-3.52) |
| Poverty to Income Ratio | 0.95 (0.89-1.02) | 1.04 (0.82-1.31) |
| Education Level |  |  |
| College Graduate or Above | 0.52 (0.37-0.74) | 0.59 (0.26-1.33) |
| High School Graduate/GED or Equivalent | 0.84 (0.63-1.21) | 0.76 (0.26-2.21) |
| Some College or AA Degree | 0.72 (0.55-0.94) | 0.66 (0.33-1.29) |
| Marital Status |  |  |
| Never Married | 1.00 (0.74-1.35) | 0.94 (0.52-1.69) |
| Widowed/Divorced/Separated | 0.98 (0.77-1.25) | 1.44 (0.60-3.46) |
| Clinical Predictors |  |  |
| BMI (kg/m^2^) | 1.03 (1.02-1.05) | 1.00 (0.97-1.03) |
| Total Cholesterol (mg/dL) | 1.00 (1.00-1.01) | 1.00 (1.00-1.01) |
| Current Smoker | 1.28 (1.00-1.64) | 1.87 (0.99-3.55) |
| Statin Use | 0.92 (0.62-1.35) | 0.47 (0.10-2.35) |
| Models were adjusted for age, sex, race/ethnicity, education, employment, insurance, marital status, BMI, diabetes, and CKD | | |
